# Supplementary material for: Innate visual attraction in wood ants is a hardwired behavior seen across different motivational and ecological contexts
Source: Insectes Soc. 2022 Jun 27;69(2-3):271–7. doi: 10.1007/s00040-022-00867-3 (PMC9314291; doi:10.1007/s00040-022-00867-3)
Supplement: Supplementary file 1 — Supplementary file1 (PDF 185 KB) [file 40_2022_867_MOESM1_ESM.pdf]

Innate visual attraction in wood ants is a hardwired behavior seen across different motivational and ecological contexts

Cornelia Buehlmann & Paul Graham

School of Life Sciences, University of Sussex, Brighton, BN1 9QG, UK

Corresponding author: cornelia.buehlmann@gmail.com

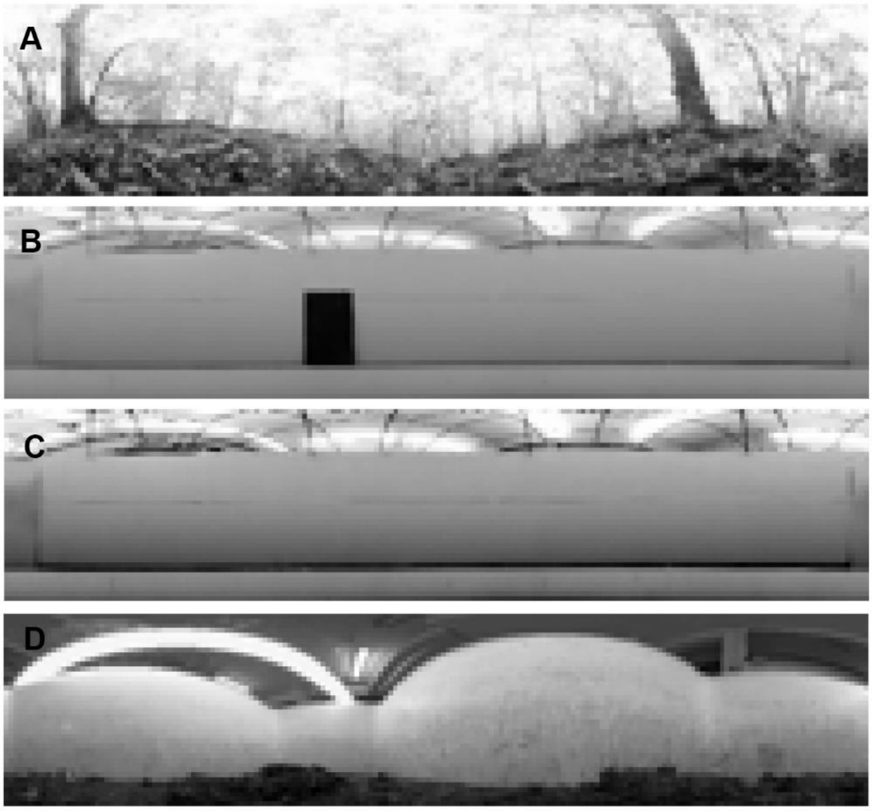

Supplementary Fig 1. Panoramic “ant’s eye” pictures from a natural wood-ant habitat in East Sussex (A), experimental setup with the visual cue (B), experimental setup with no visual cue (C) and lab nest environment (D). Panoramic images were taken with a Kodak Pixpro SP360 4K and processed in Matlab. 360° view is shown with 4° resolution.

Supplementary Table 1.1. Heading directions

	Conditions	Sample size	Mean (°)	Confidence interval	Rayleigh test (p value)
Visual cue	Foragers unfed	90 ants	0.6	354.4 / 6.731	<< 0.001
	Foragers fed	50 ants	350.2	340.6 / 359.7	<< 0.001
	Males	108 ants	343.3	335.8 / 350.8	<< 0.001
No visual cue	Foragers unfed	32 ants	n/a	n/a	0.27
	Foragers fed	22 ants	206.6	141 / 272.2	0.0468
	Males	55 ants	174.5	134.9 /214.0	0.0015

Supplementary Table 1.2. Heading directions: Comparisons

	Pairwise comparisons	Watson Williams tests (p value)
Visual cue	Foragers unfed vs Foragers fed	0.237 (a)
	Foragers unfed vs Males	0.021 (a)
	Foragers fed vs Males	0.463 (a)
No visual cue	Foragers fed no visual cue vs Foragers fed visual cue	<< 0.001
	Males no visual cue vs Males visual cue	<< 0.001

(a) Bonferroni corrected significance level of p = 0.0167.

Supplementary Table 2.1. Walking speed and path straightness

	Conditions	Sample size	Median (Walking speed, cm/sec)	Median (Path straightness)
Visual cue	Foragers unfed	90 ants	3.3	0.37
	Foragers fed	50 ants	2.5	0.36
	Males	108 ants	4.0	0.77
No visual cue	Foragers unfed	32 ants	2.8	0.19
	Foragers fed	22 ants	2.1	0.16
	Males	55 ants	4.1	0.51

Supplementary Table 2.2. Walking speed and path straightness: Comparisons

	Pairwise comparisons	Walking speed, Kruskal Wallis with Mann Whitney test and Bonferroni correction (p value)	Path straightness Kruskal Wallis with Mann Whitney test and Bonferroni correction (p value)
Visual cue	Foragers unfed vs Foragers fed	< 0.001	1
	Foragers unfed vs Males	0.02	< 0.001
	Foragers fed vs Males	< 0.001	< 0.001
No visual	Foragers unfed vs Foragers fed	0.38	1
	Foragers unfed vs Males	0.003	< 0.001
	Foragers fed vs Males	<0.001	< 0.001
